# Observations on Delirium Tremens

**Published:** 1829-01

**Authors:** John Smith


### DELIRIUM TREMENS.

*Observations on Delirium Tremens.* By John Smith, Esq. Read at the Westminster Medical Society.

It has generally been noticed that this disease attacks persons who have been addicted to excessive drinking, either malt or spirituous liquors; nor do I think there is a case on record in which the *true symptoms* of delirium tremens existed, that might not be traced to some excess of this description. It might be presumed, however, that in some peculiar constitutions this morbid disposition might be induced by a comparatively trifling irregularity; though I doubt whether it has ever been found to occur in a truly abstemious habit.

When an individual has once ruined a good constitution by drinking, I think we are justified in looking upon him as predisposed to this disease, although, perhaps, the exciting cause for the time be withdrawn. A justly esteemed author, on this subject, says, that "when habits of intemperance, especially in the use of spirits, are once established, it is very difficult to break away from such indulgences;" and he gives as an example the case of a lady, who was attacked with a train of symptoms very similar to delirium tremens. On questioning her friends privately as to the probability of her being in the habit of enjoying the pleasures of the bottle, the idea was laughed at; but, during her illness, a large bottle of the compound tincture of lavender was found in a drawer, with a liqueur glass, stained with the tincture, by the side of it; which at once proved that the habit had gone far beyond the observations of the family.

As an additional testimony in support of the opinion that this disease occurs particularly to those in the habit of drinking spirits, may be adduced the frequent occurrence of it in particular parts of the coast, where smuggling is carried on to great extent, and ardent spirit obtained at a comparatively low price. It is also extremely prevalent in some parts of America, where spirit may be had equally cheap.

It is, I believe, a generally received opinion among those who have written on this disease, that it depends upon a peculiar state, or affection, of the sensorium commune; but in what particular state, or affection, they do not sufficiently explain. Nor do I believe it to be an enigma very easily solved, as the symptoms during life are sometimes excessively perplexing, and the post-mortem examinations reveal but little, and that little very often extremely unsatisfactory.

I recollect the post-mortem examination of a sailor at Deptford, who was supposed to have died of delirium tremens. After a very careful and minute examination of the head, the morbid appearances found were slight opacity of the tunica arachnoides, an increased exudation between it and the pia mater, and a larger quantity than natural in the lateral ventricles. But I am convinced I have seen these morbid appearances repeatedly in the heads of individuals who have been accidentally killed, and who, had it not been for the accident, would probably have lived for years.

It not unfrequently happens, according to Dr. Sutton, that this disease is followed and complicated with symptoms of apoplexy, paralysis, coma, and mania; which he considers presumptive proofs that this disease may, in its fatal termination, be connected with some of those consequences which attend other affections of the head, and would not, in examination after death, be surprised to find water between the membranes, and turgescence of the veins of the head; though this opinion may claim our particular attention, it would, perhaps, have been more satisfactory had he adduced cases in justification of it.

It has been stated that this is a disease depending on a peculiar state of the animal system, brought on by excessive indulgence in spirituous or other exciting liquors.

As far as I have been able to judge, the symptoms seldom or never commence while the patient is in the daily habit of swallowing the poisonous draught, but that they occur more especially when the excitement is discontinued. In three cases which have come under my own immediate observation, and in others which I have collected, I find that the first development of the attack generally took place when the usual stimulating liquid had been left off, either from the individual's not possessing therewith to purchase it, or it had been discontinued from slight ailment or accident; which last occurrence is sometimes the cause of this delirium being mistaken for that very peculiar condition of the nervous system which occasionally follows very severe injuries, and has therefore received the name of "Delirium Tramaticum."

The following are the symptoms which I have generally found to precede an attack of delirium tremens: We will suppose the person to be in the habit of drinking daily some considerable quantity of stimulating and intoxicating liquid: for a few days after a greater excess than usual, he complains of a sense of depression or sinking at the pit of the stomach; he loses his appetite and relish for food, which he justly attributes to indigestion arising from irregularity; he accordingly refrains from it altogether, and perhaps takes opening medicine, and lives low. For the next day or two, these symptoms increase, the fluttering and depression become excessive, and his friends notice his manner to be more nervous and irritable. These symptoms are succeeded by wanderings in intellect, peculiarities in behaviour, and restless nights. The tremor, so characteristic of the disease, becomes very manifest; the bowels, if open, become costive, and the secretions of the body generally altered. If sleep has continued up to this time, it is now banished, and the delirium, particularly at night, becomes furious.*

* From the following case, it appears that the brain may be almost immediately affected by the speedy absorption of spirituous liquors. We give the extract from Dr. COOKE's work on Nervous Disorders, vol. i. page 221.

"I am informed by Mr. Carlisle, that a few years since a man was brought

These I have mentioned as some of the most prominent symptoms. I have refrained from alluding to the pulse and tongue in the first stages, as I have, in my small experience, found them to vary considerably in different cases.

With respect to the resemblance of this disease to idiopathic phrenitis, which some authors and many others have contended for, it may be observed that this is likewise an idiopathic disease, commencing with very little previous fever, and the delirium attended with much wandering irritability, restlessness, and exertion.

In these symptoms, the diseases are certainly somewhat similar: but in phrenitis you have great intolerance of light, pain in the head, a dry and furred tongue, hot skin, flushed countenance, and suffused eye.

Now, in delirium tremens there is no very great intolerance of light, though its stimulus may serve to keep up the excitement. There is a constant tremor, which is not a necessary attendant on phrenitis, but is even looked upon as something accidental when it does occur. The tongue is generally moist, though I have seen it occasionally white and furred in the centre; and sweating is a constant attendant on delirium tremens, but is wanting in phrenitis; or, if it be present, it is considered a very favorable symptom.

It has been noticed that this disease has sometimes been mistaken for mania. If I might be allowed to offer an opinion, I should say that it more nearly resembled this state of the nervous system than any other that I am acquainted with; but still there are circumstances which cannot fail to mark a difference between the two, and I shall mention in particular the species of delirium.

Maniacs are generally delirious on some peculiar topic, and their whole conversation is directed towards that point; and, during their paroxysms, nothing I am aware of will pacify them. But, in delirium tremens, the delirium is not confined to one subject, but about a variety of things which may have happened lately in their private affairs; and one grand characteristic distinction is, that they know perfectly well what dead into the Westminster Hospital, who had just drank a quart of gin for a wager. The evidences of death being quite conclusive, he was immediately examined; and within the lateral ventricles of the brain was found a considerable quantity of a limpid fluid distinctly impregnated with gin, both to the sense of smell and taste, and even to the test of inflammability. The liquid (says Mr. Carlisle,) appeared to the senses of the examining students as strong as one third gin to two thirds water."—This case is, of course, not introduced as one of delirium tremens. The fact mentioned, and so well authenticated, is interesting in relation to the occasional effects that may arise from excessive spirituous potations.—Editors.

is passing around them: so much so, as at times to be able to distinguish their friends from strangers.

In addition, the recollection of that which has immediately happened appears to be effaced on recovery, and their illness appears to them like a dream.

Having thus far, then, traced the history, (I own in a very cursory and imperfect manner,) I shall now proceed to the treatment of the disease, which I take to be not only the most interesting part, and calculated to excite a lively interest, but also fraught with considerable importance; especially to the young practitioner, who is bound to steer between modes of practice so diametrically opposite in their nature, and attended, as far as I have been able to judge, with very different results.

I shall consider separately the various and most essential modes of treatment which have been recommended, and illustrate them, as much as lies in my power, with reference to established facts and cases. To commence with the antiphlogistic treatment.

It is sufficiently evident, from the numberless cases which we meet with recorded in all the different periodicals of the time, that this disease has been, and still is, frequently attempted to be cured by those methods which are commonly resorted to in the most serious and alarming diseases of the head.

They have but one common object, which is to remove the cause on which it has been at different periods supposed to depend: viz. turgescence of the venous system, increased action in the arterial system, effusion, or extravasation. How far these opinions are correct, it will be our particular object to determine.

It is needless to mention that the usual means adopted to counteract these supposed evils, by the followers of the antiphlogistic system, have been bloodletting (both general and topical), blistering, and purging. I shall offer a few remarks upon the efficacy of these measures, as far as delirium tremens is concerned; then pass on to the consideration of general and diffusible stimuli; and, lastly, to that of opium.

In some cases of this disease, the symptoms would appear to be ushered in with an uncommon degree of excitement, quite sufficient to mislead a young practitioner, and induce him to bleed largely and repeatedly; especially when he observes his bloodlettings followed by what appears to him to be temporary relief, but which would be known to an individual of experience to be a fallacy, and the forerunner of a depression, which is frightful in its appearance, and often most alarming in its consequences.

I will relate the particulars of a case which fell under my own observation a short time back.

I was requested by a surgeon, (and a surgeon to an hospital, a man of very considerable practice,) to go to bleed a man. The patient was a publican, a man who, his friends allowed, had been in the habit of drinking two or three quarts of porter, with half a pint to a pint of spirit, daily. He had been delirious three days, occasionally furious, and had been bled, by order of the surgeon, twice before. When I saw him, he was sitting up in bed, with two or three persons to prevent him from getting out. He was bathed in perspiration; had a rapid, but small pulse; a moist tongue; constant tremor and delirium. I immediately proceeded to bleed him, which he allowed me to do without resistance. I took, according to my directions, sixteen ounces. I was sorry I neglected to count the pulse previous to the bleeding, but immediately afterwards it became so rapid that, after repeated attempts, I was unable to do so. After waiting ten minutes or so, I left him, and was rather astonished to hear the next morning that the patient was dead, and that the fatal event took place exactly three hours from the bleeding. The nurse in attendance told me that, after I left him, they laid him down on the pillow; he remained very quiet, in fact, they thought he was passing off to sleep; in consequence of which, they did not disturb him for two hours, at which time they found him gasping for breath, and he died without uttering a syllable.

The case has made such an impression on my memory, I can never forget it. I cannot help thinking, from subsequent observation, that this was a confirmed case of delirium tremens, and that he would probably have recovered had it been treated in a different manner.

I shall now consider what further has been said on the treatment of bloodletting. If the patient be of a strong plethoric habit, who is liable to determination of blood, and has pain in the head, a suffused eye, a quick and full pulse, and loaded tongue,—in fact, symptoms showing that the case is complicated with apoplexy,—there can be no doubt of the propriety of withdrawing blood, and that directly. But serious deliberation will be demanded before another copious abstraction of blood is made. This I consider, however, an extreme case: such are not the general symptoms of delirium tremens. On the contrary, you find the disease ushered in with a quick but small pulse; no pain in the head, but rather a sense of lightness; no suffusion of the eye; but a moist tongue, and such a general state of excitement and tremor that even a trifling bleeding would frequently throw the patient into the greatest state of depression.

The following passage is taken from Dr. SUTTON's pamphlet on this subject: "When bloodletting has been employed, and principally relied on, I have observed a fatal termination of the disease in almost every case, though the indication, as to habit, for its use appeared strong and decisive. And I have witnessed the cases of this disease to be always, on this account, the most rapidly fatal in robust and plethoric persons, where bloodletting was most used, without the aid of opium."

From the foregoing observations, and the opinions of men who have had considerable experience in this peculiar disease, I cannot think that bloodletting is a practice imperatively called for; but at the same time we must allow that its use occasionally paves the way for the exhibition, and materially assists the action of other remedies.

With respect to blisters, from the little experience I have had, instead of being of service, I should say they were decidedly injurious, and serve to keep up an irritation in the system, which we are, by every means in our power, endeavouring to lessen.

As to purgatives, I conceive the exhibition of them can never be looked upon as a cure for the disease; though the timely use of them will serve two good ends, viz. to clear out the bowels from any irritating contents, and aid the effects of other remedies.

With regard to the use of cordials and diffusible stimuli, such as brandy, ammonia, wine, turpentine, &c. I have seen very few cases in which they have been alone relied on. It would appear, however, that the peculiar condition of the nervous system which follows compound fractures and other severe accidents, and in cases of erysipelas of the head especially, which have been treated in the acute form by very decided antiphlogistic means, are, in the stage of the scaling, liable to a train of symptoms very much resembling those of delirium tremens, and are much benefited by the exhibition of cordials and stimuli, particularly that of opium: several instances of which have lately occurred at St. George's Hospital, and tend much to confirm this opinion.

I shall now speak more directly of opium; and I do so with considerable pleasure, as I have at this moment the notes of two severe cases successfully treated by its free exhibition.

It cannot be anticipated that a disease so seriously connected with the general functions of the brain, should be universally conducted to a favorable termination; but it must afford infinite pleasure to an individual who feels interested in his profession, when he finds the patients of the present day, who are afflicted with this most peculiar disease, recover in the majority of ten in twelve, under the free administration of this most useful remedy.

In the consideration of opium there are several points which present themselves for our observation: viz. the proper time, the mode of administering it, and the relative or proportionate dose.

With due submission, I beg to make the following remarks: Conceiving it to be a pretty well established fact, that, if the patient once falls into a profound sleep, there is a very great chance, almost amounting to a certainty, that he will recover, or at all events be very much benefited; I say, if this opinion be correct, and accords with the general experience, the exhibition of opium is determined on, and it only remains for the severity of the symptoms, and the state and condition of the patient, to be considered.

A question, however, arises as to the proper time for giving opium, and the proportion or dose, and the best form of this remedy. Some practitioners have argued it should be given immediately the symptoms of delirium are discovered, and in large and frequently repeated doses, till the desired effect is produced.

I am certain that the system in this condition will bear a much greater quantity of opium with impunity, than it will under other circumstances or in health; but, at the same time, the practitioner should be very firm in his opinion before he ventures to pour in dose after dose of a drug so energetic in its action. At all events, this observation should have some weight in laying a restraint upon its use in any other circumstances than those of strong and decided proofs of the existence of the disorder.

The following are extracts from my notes of two interesting cases that have lately occurred in the practice of St. George's Hospital.

Case I.—John Sivier, æt. thirty-five, assistant to a wholesale brewer; of a naturally good constitution, though of late impaired by excessive drinking, principally malt liquor.

September 18th.—Complains of acute pain in the lower part of the abdomen and region of the bladder, coming on in paroxysms, and extending along the urethra to the glans penis; there is incapability of voiding his water, though he is constantly making efforts so to do; pulse ninety-six, and full; bowels confined; tongue furred; skin hot; no pain in head, or sense of shiverings. A full-sized catheter was introduced without the least difficulty, and about ten ounces of very high-coloured urine withdrawn.

Hirudines xv. reg. vesicæ.—Hydr. Subm. gr. vj.; Antim. gr. iij. statim. Haust. Sennæ post horas sex.

19th.—On the whole, better. Medicines have acted freely on the bowels; and he has voided urine in small quantities during the night, which is still high coloured, but no apparent admixture of blood.

Haust. Salin ℥iss.; Magn. Sulph. ʒi.; Vin. Ant. Tart. m.xx. quartis horis.

20th.—On visiting him this morning, a different set of symptoms presented themselves. His wife states that during the night he gradually became restless and wandering; towards morning, quite delirious, talking incoherently, and requiring the assistance of two or three persons to keep him in bed. At present he lies with his eyes wide open, accompanied by a maniacal wildness; there is constant motion of the head and arms, and if asked to give his hand, he does so with a convulsive effort; many of the muscles, especially those of the forearm, are in firm contraction, and there is universal tremor of the whole body; pulse 100, small; bowels open; tongue moist and white; no acknowledgment to pain in the head, but answers quickly that it is a sense of lightness, and wanders to another subject. On questioning his wife, she said that for two or three years he had been in the habit of drinking a great deal of malt liquor, but for the last week or fortnight has taken little or nothing, on account of the pains which he suffered in the situation of his bladder. Complained very much, yesterday afternoon, of beating of the heart, fluttering, and oppression about the præcordia, previous to the commencement of the delirium.

Hyd. Subm. gr. v. statim. Pil. Opii gr. i. secundis horis. Mitte xij.

21st.—Has passed a very restless and bad night. The general symptoms, on the whole, are much aggravated; pulse 120, very small; perspiration profuse; nervous excitement and irritability excessive.

Pil. Opii gr. i. omni hora. Injectio Amyli cum Tr. Opii m. xxx.—Mitte viij.

Vespere.—Excitement and tremor have continued during the day, without intermission. He has taken the pills regularly, but there has been no disposition to sleep.

Pil. Opii gr. ij. omni hora, mitte iij.

22d.—Having passed another night equally bad with the preceding in respect to delirium, he was brought into the hospital, and placed under the care of Dr. Hewett.

Repr Injectio cum Tr. Opii ʒi. Sumat Liq. Opii Sedativ. ʒss. statim et repetet m. xx. per quatuor vices.—Beef tea, &c.

Vespere.—Began to doze this afternoon about four o'clock, and is at present fast asleep. The twitching of the muscles continues, but is much less. Took altogether thirty-five grains of opium and one drachm and a half of Liq. Opii Sed.

23d.—Has slept nearly all night, and is now quite rational and collected; still some twitching and spasmodic action of the muscles, particularly those of the forearm; pulse eighty, very soft; tongue moist, a little coated; has no pain in the head. Says that he has had two similar attacks before, and was in the hospital two years ago.

Sumat Tr. Opii m. x.; Sp. Æth. C. ʒ ss.; Mist. Camph. ℥iss. M. sextis horis.—Porter ℔ ss. bis die; beef-tea, &c.

25th.—Is much better; sleeps well. Perstat.

29th.—Has progressively improved since last report, and appears in all respects well. Omit[r] omnia.

October 6th.—Discharged cured.

Case II.—H. D., ætatis thirty-three, foreman to an extensive coach manufactory; of a stout athletic figure, and a man who has been accustomed to drink freely during his hours of work, especially ale early in the morning. Was brought to the hospital about four o'clock in the afternoon of the 19th of October. His friends give the following history of his illness:

About a fortnight ago he first complained of pains in his stomach and bowels, which he was persuaded arose from drinking so much malt liquor; he accordingly abstained from it altogether, and took nothing but food of a farinaceous description and water. After a few days, his sisters noticed his manner to have become more nervous and irritable than usual; and, on the night of the 16th, without any apparent cause or complaining of pain in his head, became rambling in his intellects, and during the night quite delirious. A medical gentleman was called in, who looked upon the case as one of phrenitis, and therefore bled him, by means of cupping, to thirty ounces, which produced fainting for half an hour; gave him a dose of calomel and an aperient draught; and directed, after its operation, to take six grains of the Pil. Hydrarg. every eight hours. The symptoms during the day somewhat lessened, and he slept for two hours; after which he was quite rational, but in the course of the night became as delirious as ever. The same gentleman saw him, and took away thirty ounces of blood from the arm, which again induced fainting for half an hour. He was then directed to take twelve grains of the Pilula Hydrargyri every eight hours; of which altogether he has taken one drachm.

October 19th, four P.M.—we first saw him: he was then sitting in a chair in the ward, with his hat on, in a most unconcerned manner. The symptoms that attracted our particular attention were, the peculiar wildness of the eyes, the great nervous excitability, universal tremor, amazing irritability of a small pulse, moist tongue; absence of acknowledgment to pain in the head or elsewhere; constant talking, though evidently incoherent; and the rapidity with which all the motions of the body were performed. The foregoing symptoms, combined with the account of the man having been a free drinker, were in favor of its being a case of delirium tremens, rather than one of phrenitis requiring further depletion. In the evening, the delirium and tremor becoming more decided, it was judged prudent to confine him in a waistcoat.

He was directed to take Tr. Opii ʒss.; Mist. Æth. ℥ss. statim. Tr. Opii ℈i.; H. Piment. secundis horis.

Twelve P.M.—For the last hour, the delirium has been excessively furious; he is almost unmanageable; continually struggling and vociferating. He has taken two doses of the mixture, but cannot be persuaded to take any more at present. He lies in a profuse perspiration; lips and tongue dry; pulse quick and small, not easily counted, (perhaps 140;) has neither passed water nor motion since admission. The peculiarity of the delirium in these cases, so often noticed, that, even in the most furious paroxysms, they know perfectly well what is passing around, was perhaps never more strongly marked than in this case.

Towards morning he took Tr. Opii ʒ ss.; Mist. Ætheris ℥iss.

October 20th, eleven A.M.—The delirium and tremor continue, but are less violent. About an hour ago, he allowed the nurses, with the assistance of two or three men, to change his waistcoat and shirt, which was irritating a blister at the back of the neck, applied previous to admission. He was afterwards removed to a fresh bed; his tottering, uncertain step was particularly noticed. Having been seen by Dr. Young, he was directed to take ʒss. of the Tinct. Opii every two hours in Mist. Camph. He has passed plenty of water in the bed.

Six P.M.—The delirium still continuing, he was removed to a lower apartment of the hospital, being fearful he might again disturb the ward during the night. He has taken two more doses of his mixture, making in the whole four drachms two scruples of the Tinctura Opii taken since his admission.

Eleven P.M.—He has been very quiet since his removal, and began to doze, for the first time since the 18th, about eight o'clock this evening, and is now fast asleep. Bowels have not acted since admission; pulse 110 and small, counted during sleep. The nurse was directed, if he should wake up within an hour, be uneasy, or noisy, to repeat a dose of his mixture; if not, to discontinue it altogether.

21st, nine A.M.—Has slept nearly the whole night, at intervals waking up, asking for drink, then subsiding again quietly to sleep. Altogether there is a very marked change. He has passed plenty of water, and bowels have been open once; there is still slight tremor, but he is wonderfully better.—Haust. Sennæ.

Two P.M.—Has slept a good deal during the day, and is in every respect going on well. He is now perfectly sensible, and, when told about his late behaviour, listens with much concern, and hopes he has done no mischief; says that he recollects coming to the hospital, but nothing of the circumstances that have taken place since. Pulse 120, very small; tongue rather coated in the centre, and dry. The draught has acted freely on the bowels.

Sumat Tinct. Opii ℈ss.; Sp. Æth. C. ʒss.; Mist. Camph. ℥iss. M. quartis horis sum.—Beef-tea, &c.

October 22d.—In all respects improving: was rather restless in the middle of the night, but altogether slept well. The waistcoat has been taken off, and he has been removed into the ward. There is still a slight tremulous motion of the forearm, but it has nearly subsided. There is a peculiar fetor about him, which may be attributed to the mercury, his gums being slightly affected. Bowels open; tongue moist, and less furred; pulse 104, small and feeble. Only complaint is his hands, which are chafed and swoln, from the bandages of the waistcoat.

Cont^r medic. heri pres.

23d.—Going on favorably: intellect perfect; countenance natural; bowels open once; tongue rather dry; pulse 100, more steady. Takes beef-tea, and the mixture every eight hours.

26th.—He got up yesterday morning for the first time, and remained up for some time; after which, it was thought that his manner was rather more hurried, but he passed a good night, and this morning appears better. Bowels open; pulse ninety-six, steady and full; tongue moist and clean.

29th.—Has progressively improved since last report; can hold his hand perfectly steady, and is, in fact, quite well. Wishes to go to Brighton to recruit his health, previous to commencing his usual occupations.

30th.—Discharged cured.

In the recital of these cases, I have purposely refrained from making any remarks, as it might deprive them of their interest; but I think the utility of the opium must be very evident.

There are a few questions upon which I much wish for satisfactory information. Does bleeding increase the delirium in these cases? Does it lessen the quantity of opium necessary for its cure? What is the best form of giving opium: in the way of tincture, the gum, or the liq. opii sedativus? and what is the result of experience with respect to the exhibition of suppositories? I understand it is M. Dupuytren's opinion that a small quantity of opium administered per anum is generally more powerful in its effects over the system than the same, or even a larger, quantity given by the stomach. His reasons are, that the opium undergoes no change in the rectum as to its actual composition; whereas, in the stomach, it is very soon altered, to a certain degree, by the gastric juice, and the other substances contained therein. I conceive this to be a point worthy consideration, and, if it be true one of great practical importance.

I certainly do think, as far as my experience goes, that the use of suppositories is very frequently neglected in this country; whereas, it is found of very great service, and in general use, on various parts of the continent.

---

